# The relative contribution of intraspecific variation and species turnover to the community-level foliar stoichiometric characteristics in different soil moisture and salinity habitats

**DOI:** 10.1371/journal.pone.0246672

**Published:** 2021-02-17

**Authors:** Yelike Tusifujiang, Xueni Zhang, Lu Gong

**Affiliations:** 1 College of Resources and Environment Science, Xinjiang University, Urumqi, China; 2 Xinjiang Key Laboratory of Oasis Ecology, Xinjiang University, Urumqi, China; Qinghai University, CHINA

## Abstract

**Background:**

Trait-based approaches have been used to demonstrate the responses of plant functional traits to environmental change may manifest both among- and/or within-species. However, how community-level foliar stoichiometric characteristic variations respond to aridity and salinity is still not well-known.

**Methods:**

We calculated community weighted means (CWMs) and non-weighted means (CMs) of foliar C, N, and P concentrations (and their ratios) in a dryland plant community respond to high (HSW) and low soil moisture and salinity (LSW). Based on a sum of squares decomposition method, we determined the relative contributions of intraspecific variation and species turnover in both HSW and LSW habitats.

**Results:**

The CWMs of foliar C, C:N and C:P, and CM of N in the HSW habitat were significantly greater than those in the LSW habitat. The trait variations in two habitats were mainly driven by intraspecific variation, and its contribution to trait variation mostly declined with the decrease of soil moisture and salinity. The CWMs of foliar C-related stoichiometric characteristics were mainly dominated by species turnover in both habitats. Moreover, the contribution of species turnover to C and C:P variations showed an increasing trend in the LSW habitat. For CWMs, negative covariations between intraspecific variation and turnover occurred in HSW and positive covariations (except N:P) occurred in LSW; however, CMs were generally positively correlated in both habitats.

**Conclusions:**

The intraspecific variation declined as drought stress intensified, which indicates that the adaptability of desert plants declined when the stress changed from salinity to aridity. The total variation of C-related traits in both habitats were mainly dominated by species turnover. These findings highlight the importance of intraspecific variation in driving desert plant response of community functional composition to salt stress, and the joint role of intraspecific variation and species turnover in resisting drought stress.

## Introduction

How plant functional traits vary across environmental gradients is a primary question of trait-based community ecology [1, 2]. The last two decades have seen a heightened interest in functional trait research [3–6], with a focus on how intraspecific variation (ITV) and species turnover (SPT) contribute to community-level trait variation [7]. This research provides new perspectives into the mechanisms underlying plant community assembly and environmental responses.

Studies of functional trait variation provide meaningful information on plant responses to environment gradients [8, 9]. Changes in community-level functional traits across environmental gradients are determined by intraspecific variation, interspecific variation, and their interaction [10, 11]. Intraspecific trait variation is the overall variability of trait values and trait syndromes expressed by individuals within a species [12]. ITV is driven by both phenotypic plasticity and genetic variation [13]. A higher contribution of ITV to community-level variation implies greater adaptive ability of individual plant species and greater community resistance to disturbance [14]. Species turnover is derived from changes in community composition [15]; if the majority of variation is interspecific, this implies lower plant community resistance to environmental change [10]. Previous researchers have mainly considered species turnover, assuming that functional trait variations among species are much larger than those within species [1, 16] and ignoring the role of intraspecific variability [17–19]. However, the relative importance of ITV to community trait variation can be larger than that of SPT in certain habitats [12, 20–22]. Especially to sub-dominant species, ITV may merit greater attention [23].

Research that predicts plant community responses to environmental change by considering both ITV and SPT has become increasingly important in recent years [10, 12]. Whether community trait variation is driven by SPT or ITV may depend on trait type, spatial and temporal scale, various community characteristics, and even the method used to calculate these effects. To date, researchers have shown varying degrees of SPT and ITV influence in different environments. In Tibetan alpine meadows, ITV drove community-level trait variation when under grazing [15]. Similarly, in a semi-arid grassland, ITV contributed more to the stoichiometric response to mowing and fertilization treatments than did SPT [14, 24]. In contrast, Lepš *et al*. found that variation in community foliar carbon content was dominated by species turnover in semi-natural meadows [5]. However, in a natural forest ecosystem in central Chile, altitudinal changes in a widespread tree species induced only through ITV [25]. Conversely, Kichenin *et al*. showed that SPT dominated variability in alpine and subalpine plant communities along altitudinal gradients in Southern New Zealand [10]. Suffice it to say, the sources of variation in community functional traits are highly labile.

Different functional traits respond differently to environmental change [26]. Thus, the traits included directly influence the source of community trait variation identified by a study. In a short-term fertilization experiment, variation in plant height was driven by ITV [22]. In another nutrient-addition experiment in alpine meadows, variation in plant height was instead driven by SPT [6]. As important chemical leaf traits, foliar C, N, and P concentrations, as well as their ratios, can reflect plant functional responses to environmental change [5, 10]. Foliar stoichiometry tends to have greater ITV than morphological traits [27]. At the species-level, stoichiometric traits respond quickly and sensitively to environmental changes [28]. However, community-level responses remain poorly understood, as well as how ITV and SPT contribute to these responses [13, 29].

Community weighted trait means (CWMs) and non-weighted means (CWs) provide complementary information in assessing the role of ecological drivers of plant community structure [30]. Comparing CWM to CM is especially useful in assaying the effects of dominant (CWM) and subdominant species (CM) in response to environmental change [31–33]. Comparing these two types of means, Volf *et al*. found that, when using CM, intraspecific variation accounted for more of the variation in specific leaf area [22]. Both CMs and CWMs should be calculated simultaneously for communities with sparse plant species, such as desert plant communities. Although such analyses exist for forest and grassland communities in humid and sub-humid areas, assessments of community trait variation are rare in arid regions [34].

Dryland plant communities are generally low-density and suffer from multiple stressors, such as drought, salinization, and low nutrient availability [35, 36]. Previous works have shown that soil water content and salinity are crucial environmental factors affecting desert plant diversity [37]. However, little is known about how community traits differentiate in response to variation in soil moisture or salinity, and the relative roles of ITV versus SPT. The Ebinur Lake National Wetland Nature Reserve is located in the southwestern Junggar Basin, a part of the arid region in Northwestern China. The area is home to typical desert plant communities, the species distributions and diversity of which shift along moisture and salinity gradients. There are significant differences in foliar nutrient content among regional plant species [38], but the reasons underlying such differences are not yet known. Therefore, by analyzing variations in the foliar stoichiometric characteristics of desert plants and their responses to different soil moisture-salinity habitats, we addressed the following three questions: (1) Do the stoichiometric traits of desert plant communities change in sync with soil moisture and salinity? (2) To what extent do ITV and SPT contribute to community stoichiometric responses to soil moisture and salinity? (3) How does the relationship between SPT and ITV respond to different soil moisture and salinity habitats?

## Materials and methods

### Study area

**Fig 1:**
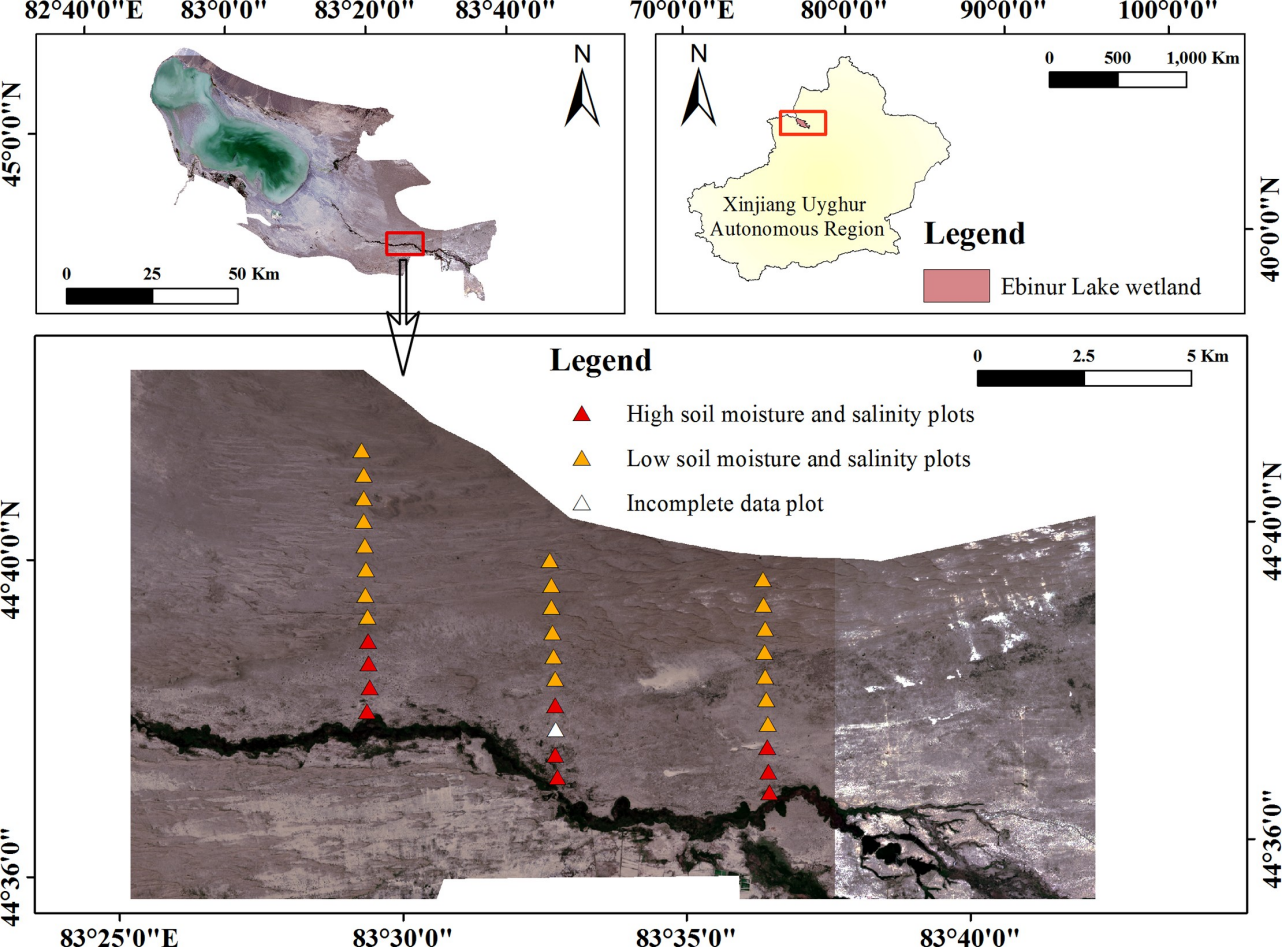
Location of the study area and landscape of Ebinur Lake wetland. Red triangles represent high soil moisture and salinity plots, orange triangles represent low soil moisture and salinity plots, white triangle represents incomplete data plot.

This experiment was conducted in the Ebinur Lake National Wetland Nature Reserve (44°30′-45°09′N, 82°36′-83°50′E) in the southwestern part of the Junggar Basin in Xinjiang, China (Fig 1). The wetland serves as a collection point for salt and water in the middle of the Basin. The study area is characterized by a typical continental arid climate, with high mean annual evaporation (>1600 mm/yr) and low precipitation (105.17 mm/yr) [35]. The Aqikesu River is one of the water sources of Ebinur Lake, and has relatively high soil moisture and salinity along the river banks that gradually decrease with distance from the river. The abundance and composition of riparian plant communities vary with soil moisture and salinity. Specifically, *Aeluropus pungens*, *Haloxylon ammodendron*, *Phragmites australis*, *Populus euphratica*, and *Tamarix ramosissima* grow near the river, while *Agriophyllum squarrosum* and *Calligonum ebinuricum* grow further from the river [39]. The study area is our field observation and research station for desert vegetation, and no permission is required to enter the field site to perform field vegetation surveys.

### Data collection

From July to August 2013, at the northern bank of the Aqikesu River in the Wetland Reserve, three transects were established perpendicular to the river channel and 5 km apart. The transects were not subject to human disturbance, and soil moisture and salinity gradually decreased along the transects with distance from the river. Along each transect, ten to twelve 10 m × 10 m plots were established (n = 32 total), with 0.5 km between plots. The numbers of all species and individuals were recorded in each plot, and 10 g leaves from the mature canopy of each species, with no pests, diseases, or no mechanical damage were sampled at the plot level and brought back to the laboratory for drying. These foliar samples were first oven dried at 70°C for 48 h to a constant weight and then ground to powder using a pulverizer (FW100, 24000 r/min, 60–180 mesh) for later analysis of C, N, and P content (g/kg). Foliar C content was obtained using the potassium dichromate-sulfate oxidation method. Foliar N content was assessed with the Kjeldahl method (with H_2_SO_4_ used to accelerate digestion). Foliar P content was quantified by first digesting with nitric and perchloric acids, and then using the molybdenum antimony colorimetric method. The above methods were all referred to Dong [40].

Soil samples (0–15 cm depth) were taken from five randomly selected points per plot, and then combined in a single composite sample per plot. Time domain reflectometry (TDR) was used to measure soil volumetric water content (SVWC) in the vicinity of sampling points. Soil samples were wrapped in ziplock bags and brought back to the laboratory for air drying and experimental analysis. Electrical conductivity (EC) was determined using a DDS-307 conductometer (Shanghai INESA Scientific Instrument Co., Ltd., Shanghai, China). EC was measured in an aqueous extraction (soil: water = 1:5).

### Data analysis

The divisions of the soil water and salt habitat were established using *k*-means cluster analysis.

Community trait means for C, N, and P, and the C:N, N:P, and C:P ratios were calculated in two ways for each plot: as weighted means (CWMs) and non-weighted means (CMs). The CWMs consider both species presence or absence data and relative abundance, whereas CMs only consider presence or absence data. The CWMs were calculated for each plot as follows:

$$CWM=\sum_{i=1}^{s}P_{i}t_{i}$$

where *P*_*i*_ is the relative abundance of species *i*, *t*_*i*_ is the mean trait value of species *i*, and S is the number of species in the plot.

To distinguish the relative contributions of ITV and SPT to variation in the CMs and CWMs, a sum of squares decomposition method proposed by Lepš *et al*. was implemented, where three composition parameters are calculated for each community functional trait [5]. (1) Specific averages utilize both the relative abundance and trait values of each species in plots, and thus include the effects of SPT and ITV. (2) Fixed averages use the species mean trait values across all plots in the study site, and thus include only the effects of species turnover. (3) The intraspecific variability effect is estimated by subtracting fixed average value from specific average value. These three values were calculated separately for the CM and CWM of each trait. Next, the total sum of squares (SSspecific) of the community-level trait variation of each habitat was decomposed into ‘fixed’ (SSfixed), ‘intraspecific’ (SSintra), and ‘covariation’ (SScov) components (*i*.*e*., SSspecific = SSfixed + SSintra + SScov). If SSintra and SSfixed are positively correlated, SSspecific will be higher than the sum of SSintra and SSfixed, and SScov will be positive. Conversely, when their relationship is negative, SSspecific lower than the sum of SSintra and SSfixed, and covSS will be negative. Lastly, the relative contributions of ITV, SPT, and their covariance were quantified using variance decomposition for every stoichiometric characteristic in both moisture-salinity habitats.

Community species composition was compared between the two moisture-salinity habitats using non-metric multi-dimensional scaling (NMDS) based on Bray-Curtis dissimilarity (BC) for relative abundance data. All variables were tested for the assumption of normality and homogeneity of variance using the Shapiro-Wilk test and Levene’s test, respectively. Square-root transformation was performed when data were not normal and homoscedastic. The statistical differences of both SPT and ITV in HSW and LSW habitats, as well as log-transformed (base 10) species specific means of N and P among three studies were compared using one-way ANOVA.

The cluster analysis, sum of squares decomposition, and NMDS were processed using the “stats”, “cati”, and “vegan” packages in R [41]. All figures were created using Origin2018.

## Results

### The classification and species diversity of the high and low moisture -salinity habitats

**Fig 2:**
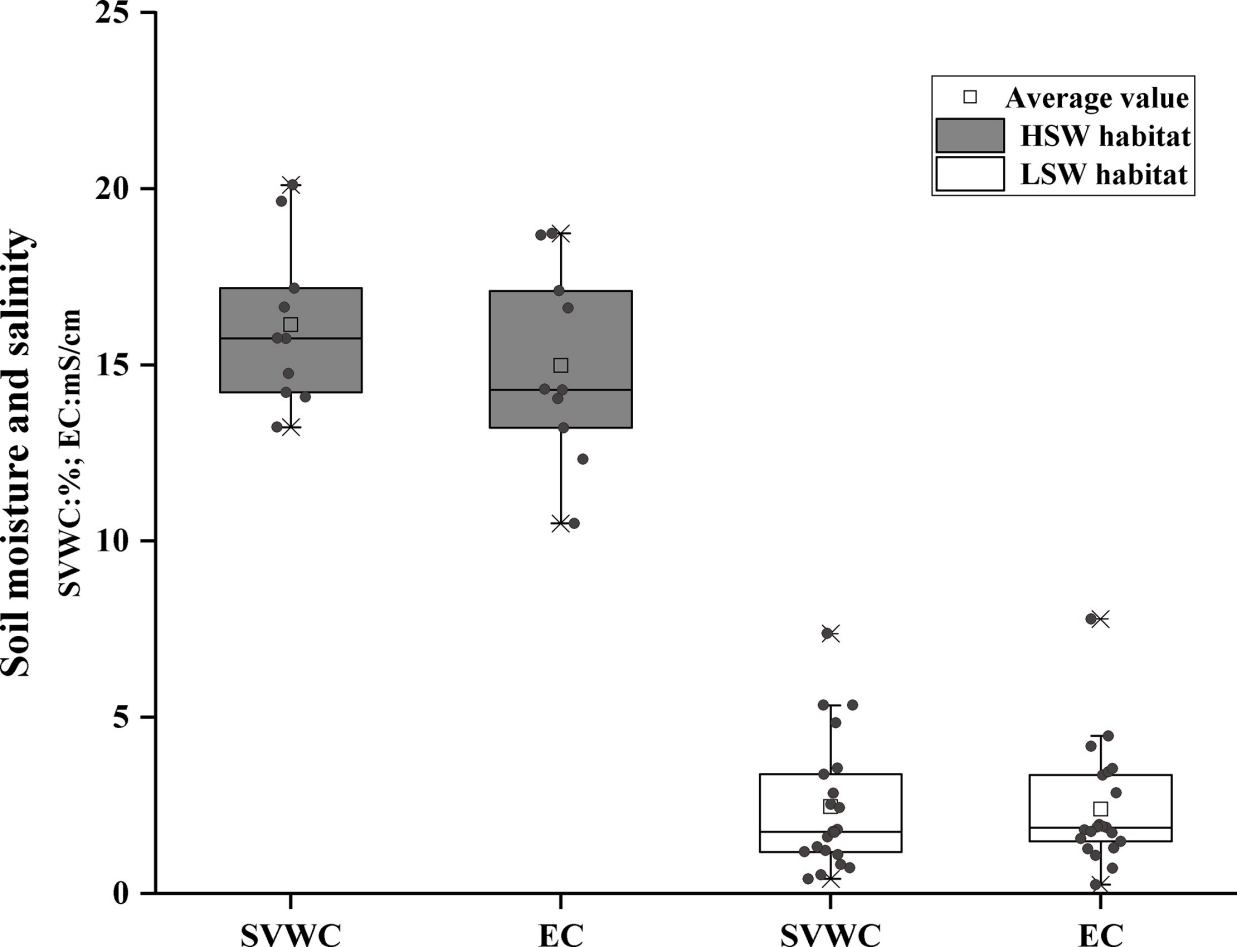
**Boxplot of the average soil volumetric water content (SVWC, gray boxes) and electrical conductivity (EC, white boxes) in high (HSW) and low soil moisture and salinity (LSW) habitat.** The black lines across the boxes are median values. The black squares above the boxes are average values.

According to clustering analysis method combining soil SVWC with soil EC, 32 soil sampling points could be divided into low moisture and salinity habitat (LSW, n = 21) and high moisture and salinity habitat (HSW, n = 11). In the high moisture-salinity group, one plot was excluded due to incomplete data (n = 10). Average soil moisture and salinity differed between the groups (*P*<0.01). We refer to the HSW as the salt stress environment due to higher soil EC, and the LSW with lower soil water content as the drought stress environment (Fig 2) [39, 42]. The species richness and evenness of HSW habitat (5.5±1.7; 0.7±0.1) were higher than that of LSW habitat (4.5±1.6; 0.5±0.3), which were not significant (S1 Table).

### Differences in foliar stoichiometric characteristics between the two moisture-salinity habitats

**Fig 3:**
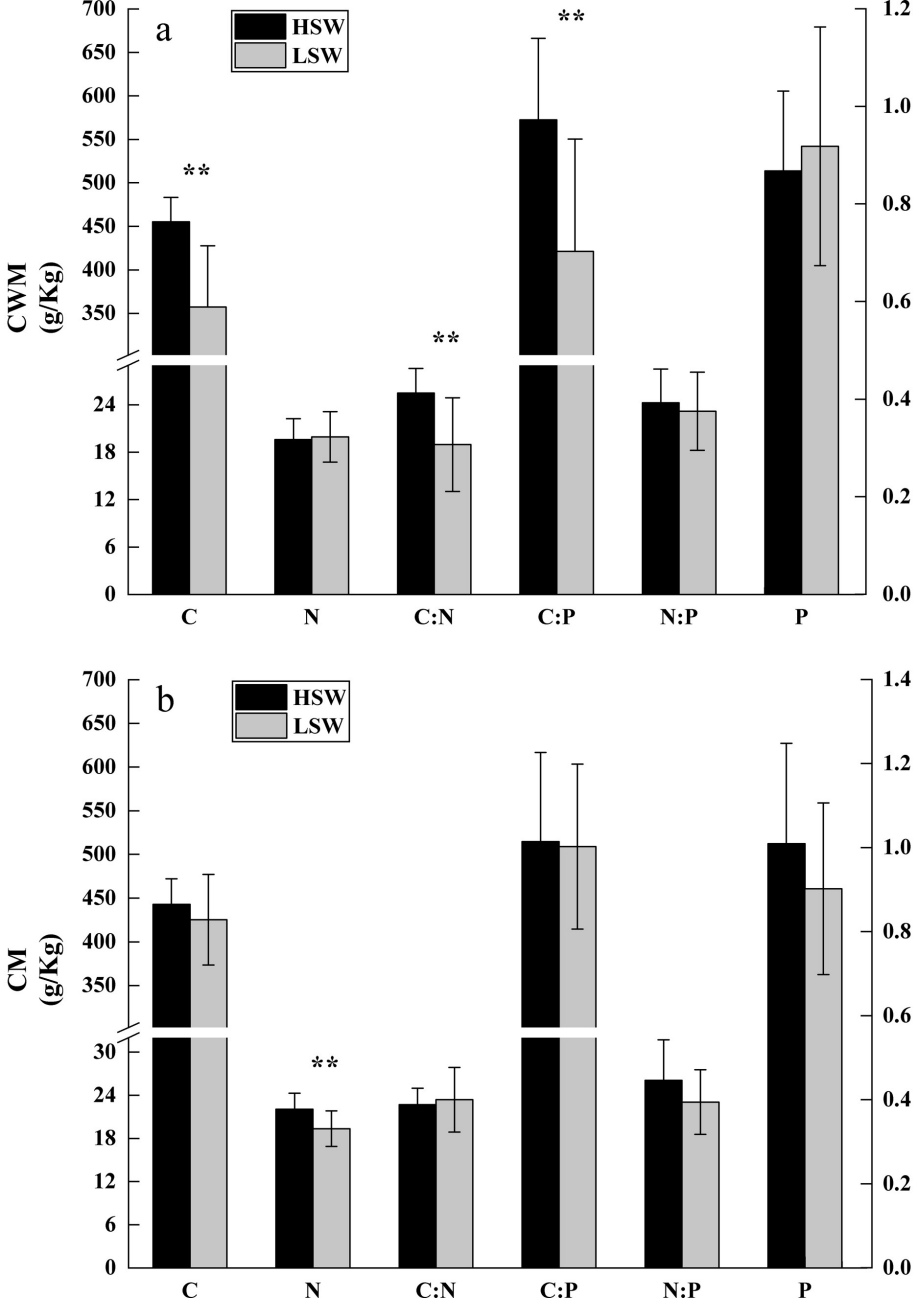
Community weighted (CMW) (left, a) and non-weight (right, b) means of foliar stoichiometric characteristics in high (HSW) and low moisture-salinity (LSW) habitats. Bar height represent the means of leaf stoichiometric characteristics and error bars show the standard error of the mean. Asterisks indicate significant differences between environments (*P*<0.01**). The C and N concentrations, and C:N, C:P, and N:P ratios are given by the vertical axes on the left. The P concentration is given by the vertical axes on the right.

Trait differences between habitats were inconsistent, and subject to the type of mean considered. Using CWMs, the foliar C content and the C:N and C:P ratios were lower in the LSW compared to the HSW habitat (*P*<0.01; Fig 3A). Using CMs, the foliar N content was lower in the LSW habitat (*P*<0.01; Fig 3B). No significant differences were found in foliar C:N, C:P, or N:P ratios between habitats (*P*>0.05).

### Contributions of ITV and SPT to trait variation in different soil moisture-salinity habitats

**Fig 4:**
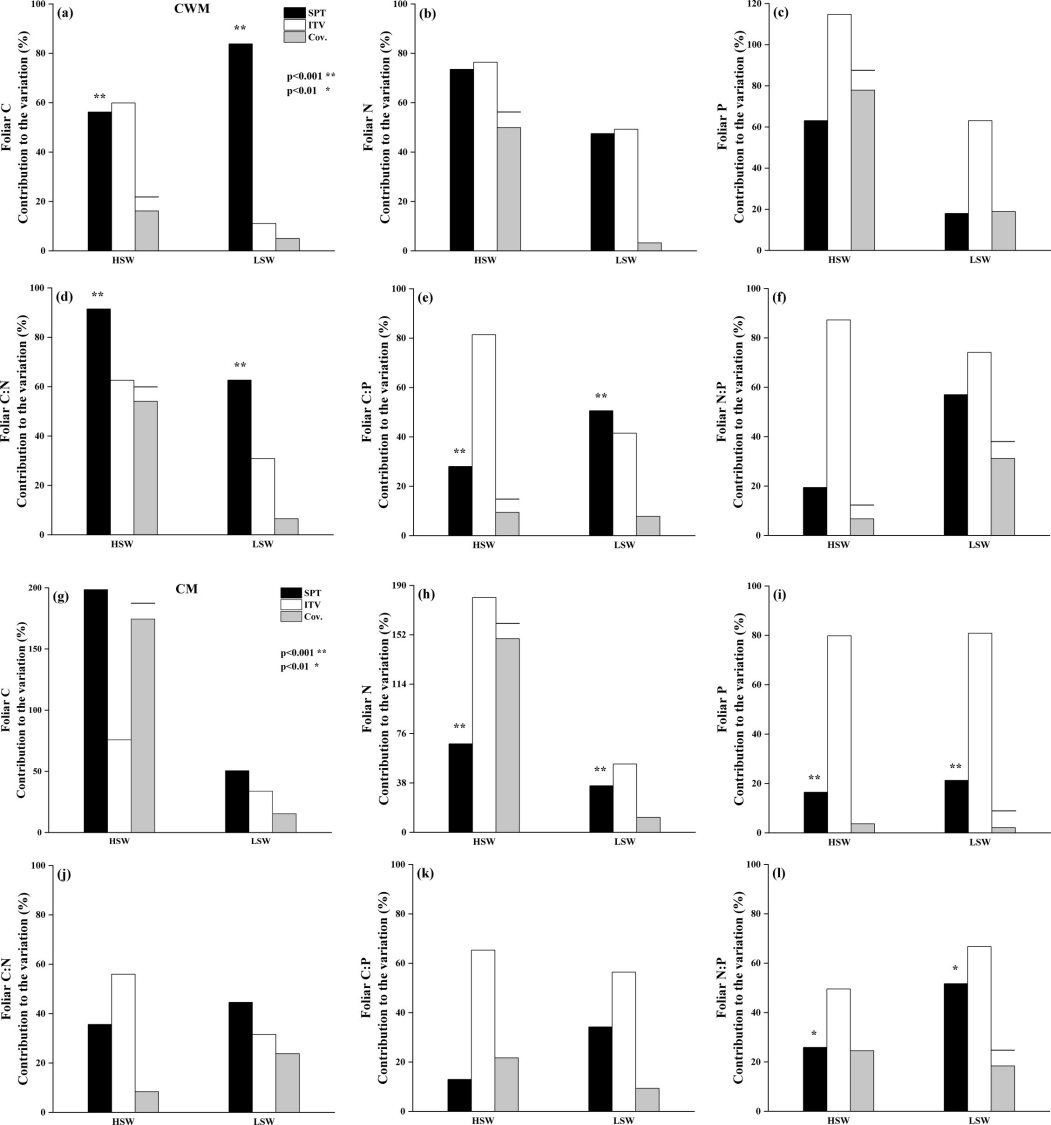
Variance decomposition in each environment for CWMs (left) and CMs (right). White bars represent the contribution of intraspecific trait variation (ITV), black bars represent the contribution of species turnover (SPT), and gray bars indicate positive covariation, whereas gray bars with horizontal lines show negative covariation. The positioning of an * within the graphic represents significance of the SPT between HSW and LSW habitats.

The total variation of all the examined leaf chemical traits was mainly driven by ITV. Using CWMs, ITV and SPT contributed relatively equally to variation in foliar C and N in HSW, as well as foliar N in LSW. However, in LSW, SPT made up more of the variation in C. We found that most variations in foliar P and N:P in two habitats were due to ITV, but the ITV made up a smaller proportion of the total variance in LSW (P 63.11%, N:P 74.16%) relative to HSW (114.8% and 87.29%) (Fig 4). Variance in C:N in HSW and LSW could both be mostly attributed to SPT, with a lower absolute proportion in LSW. C:P variation in HSW was mostly due to ITV; however, in LSW, SPT was slightly greater than ITV. In general, ITV was lower in LSW than in HSW for all trait CWMs examined. There were negative covariances between SPT and ITV of each trait in HSW, these negative covariances, however, changed to be positive in LSW, except for N:P (Fig 4).

Using CMs, ITV accounted for more variation than SPT in all traits and habitats except for foliar C (both LSW and HSW) and LSW foliar C:N (Fig 4). However, the contribution of ITV to variation in foliar N, C:N and C:P in LSW were lower, and even SPT made up more of the variation in folia C:N in LSW. In sum, contribution of ITV to trait variation were lower in LSW, except foliar P and N:P, meanwhile, contribution of SPT to trait variations were higher in LSW apart from foliar C and N. The relationships between ITV and SPT of each trait, in contrast to CWM, were mainly positive in both HSW and LSW.

## Discussion

We assessed the relative contributions of ITV and SPT to community trait variation, as well as how ITV and SPT changed with soil moisture and salinity by comparing weighted and non-weighted community trait means. We found that foliar stoichiometric characteristics had idiosyncratic responses and that the underlying mechanisms were dependent on environmental context.

### The response of community-level foliar chemical traits to soil moisture and salinity

The answer to our first question is soil moisture and salinity can significantly affect community foliar C, N, and P content—and their ratios—in desert plant communities. In this study, using CWMs, foliar C concentration was higher in the HSW environment compared to the LSW environment. The lower foliar C values in LSW may be related to drought stress because water deficits generally reduce photosynthesis and, ultimately, carbohydrate synthesis [43]. In addition, plant community composition differed between habitats, with herb abundance in the LSW habitat was more than ten-fold that of the HSW habitat (S1 Table). As foliar C concentrations are generally higher in trees versus herbs [44], this could also help explain the lower community foliar C concentrations observed in the LSW habitat. We observed significant difference in foliar N between the two habitats only when calculated as non-weighted means, and found no differences in foliar P. It is generally believed that foliar N and P concentrations are negatively affected by reductions in soil moisture [45]. The responses of foliar N and P CMs to soil environment may indicate the drought stress on common species, which may be related to desert plant adaptation to drought and salt stresses [35, 46]. Our study site was located in arid area of northwest China, the poor soil quality and high salinization degree may result in less effective nutrients obtained by leaves, and consequently lead to N deficiency. Low community foliar N and P content in this study is consistent with Luo *et al*. [47]. The communities we studied had lower average foliar P but higher N content (0.95 mg/g and 20.4 mg/g, respectively) than the global mean of terrestrial leaves (1.77 mg/g P and 20.1 mg/g N) [48], and of Chinese terrestrial leaves (1.46 mg/g P and 20.2 mg/g N) [49]. Desert plants tend to maintain high foliar N absorption capacity, which may be a survival strategy in these harsh environments [50].

The N:P ratio is often used to assess plant nutrient limitation status [51]. Plant growth is considered to be restricted by N when N:P < 14, and restricted by P when the N:P > 16 [49]. In this study, community N:P ratios were about 25 in both habitats, indicative of P limitation. The average leaf P content in this area is significantly lower than the average of Chinese terrestrial plants (*P*<0.001, S2 Table), which may further indicate general P-limitation in this area. Foliar C:N and C:P ratios reflect a plant’s ability to assimilate C when absorbing nutrients, which, to some extent, reflects its nutrient utilization efficiency [52, 53]. Meanwhile, the C:P ratio reflects plant growth rate, with a low C:P ratio indicating a faster growth rate [54]. In this study, the average C:N (22.9) was slightly higher than the global mean (22.5), while the average C:P (507) was much higher (232) [55]. These metrics suggest that these communities have high P utilization efficiency, which may increase competitiveness in arid and nutrient-poor environments [56]. Here, the weighted community C:N and C:P were lower in LSW relative to HSW, implying that community had lower carbon assimilation capacity and higher plant growth rates in the LSW habitat. This is consistent with the finding that plants tend to complete vegetative growth faster, if possible, when under stress [57].

The community-level foliar stoichiometric characteristics response to aridity and salinity differed among analyses based on CMs and CWMs. These differences suggest that dominant and sub-dominant species may respond differently to environmental change (here different soil moisture and salinity), although some patterns may remain the same for both groups of species [58]. The current study showed that the CM of N was higher than the CWM of N in the HSW environment, which was consistent with the conclusion that herbs (subdominant species) had higher foliar N content compared with shrubs and trees (dominant species) [59]. In this present research, the foliar C for CWM was significantly lower than that for CM in the LSW environment, which we believe to be caused by lower foliar C concentrations in dominant species.

### The relative contributions of ITV and SPT to community trait variation are trait-specific and dependent by soil environment

The main aim of this study was to assess the effects of ITV and SPT to community traits in two soil moisture and salinity environments. The contributions of ITV and SPT to variation in chemical traits of these xeric plant communities in different soil water contents and salinities were not consistent across traits.

Using CWMs, chemical trait variations were mainly dominated by ITV, which agrees with previous findings [27], and suggests the chemical traits of desert plants may be sensitive to changes in soil water and salinity. High ITV can confer greater resistance to environmental changes at the community level, possibly providing a flexible response to environmental change [60]. For example, we found that mean N and P of co-occuring species (e.g., *Reaumuria soongorica* and *Kalidium foliatum*) significantly changed with soil moisture and salinity. This was consistent with a study of Chinese grasslands that found community N, P, and N:P ratio variations were all driven by ITV [14]. The dominance of ITV in community-level N and P variation may indicate plant community tolerance to changes in soil moisture and salinity, and thus lead to slower species turnover. Moreover, the contributions of ITV to all trait variations were higher in HSW, indicating that foliar stoichiometric characteristics may be labile when soil salt stress intensified. Our result showed that the species richness and evenness were higher in HSW than LSW habitat. These indicated that ITV may play an important role in increasing species diversity and alleviating species loss with salt stress [61].

When SPT drives community variation, plant communities are considered to be less resilient to environmental change [10]. In our study, the community trait variations dominated by SPT were mainly related to C (i.e., C and C:P ratio in LSW, and C:N ratio in HSW and LSW). This was consistent to a study of subalpine forests of Yulong Mountain, China, where foliar C variations of different tree species were largely explained by SPT [62]. The difference in species composition between habitats—especially the abundance of herbaceous species in LSW—may be the main reason for the conversion from ITV-dominated in HSW to ITV and SPT dominated together in explaining community trait variations (S1 Fig). The conversion may strengthen the selection for drought tolerance species. For example, *Salsola arbuscula*, *Horaninowia ulicina* only occurred in LSW habitat. The relative contributions of ITV and SPT to community trait differentiation can vary by trait, along environmental gradients, and with community type [5, 10, 11, 27, 63]. In this study, we show that the strengths of ITV and SPT are also influenced by the method used to compute the community trait means.

The CMs and CWMs provide complementary information for understanding plant community traits responses to different habitats. As in CWMs, variations in CMs in this study were mainly driven by ITV, both in HSW and LSW habitat, indicating that the communities were likely robust to environmental changes [10]. Kichenin *et al*. (2013) found that ITV contributed more to non-weighted community traits than weighted community traits, and suggested a stronger effect of ITV on subdominant species than dominant ones. It may also be true that rare species have high ITV in order to adapt rapidly to changing habitats [64]. ITV of P and N:P CMs were higher in the LSW habitat. Similarly, in a semi-natural grassland of Australia, Mariotte *et al*. found that N:P ratio was more flexible for sub-dominant species, which promote the resistance of sub-dominant species to drought stress [65]. Together, these findings confirm with the notion that sub-dominant species were more resistant to drought perturbations due to higher ITV in chemical traits. Finally, except foliar C and N, the SPT of all none-weighted traits in LSW were higher than in HSW. It demonstrated that drought stress played a larger role in regulating this community composition.

The covariation of SPT and ITV may indicate that the expression of traits has similar functional roles to environmental changes [25]. Using CWM, ITV and SPT relationships changed from all negative in HSW to generally positive in LSW. Thus, the effects of ITV and SPT on community trait variability in HSW may be a result of trade-off between them. In LSW, however, ITV and SPT were showed same effects on community trait variability when drought stress intensified. Using CM, positive covariations between ITV and SPT were prevalent in both HSW and LSW, suggesting that community traits responses to environmental pressures were accomplished both by selecting adaptable species with specific functional traits, thus leading to a reinforcement of trait selection [22]. For example, in the LSW habitat, species with low N and P demands may be selected, or adaptation may be enhanced in relevant traits in low N and P environments.

## Conclusion

We found that desert plant community mean community C, N, and C:N differed between two soil moisture and salinity habitats. Community trait variations in this arid area were mainly driven by intraspecific variation, which may reflect adaption to salt and drought stress. However, species turnover played a major role in C-related trait variations (i.e., C, C:N and C:P ratios), which showed that photosynthetic productivity also differed with community composition. The interplay between SPT and ITV generally changed from negative to positive when drought stress intensified. We may speculate that the maintenance of community in salt stress habitat relied on ITV, while in drought stress habitat depended on both ITV and SPT. Drought stress had a more profound effect on the desert plant community. Overall, our study contributes to understanding the relative importance of ITV and SPT in desert plant communities and their response to salt and drought stress.

## Supporting information

S1 Table Plant habit, the number of plots which species appear, the average number of individuals per plot in the two soil moisture and salinity habitats. (DOCX)

S2 Table Statistics of leaf nitrogen (N), phosphorus (P) and N:P ratio for all species analysed by this study; Han et al. (2005) [49]; and Reich & Oleksyn (2004) [48]. *Significant difference in means of P between this study and the others (P<0.001). No significant differences in N were between studies (P>0.05).
(DOCX)

S1 Fig Non-metric multi-dimensional scaling (NMDS) ordination of community composition for plots varying in soil moisture (SVWC) and salinity (EC). Red letters are abbreviations for species only occurred in HSW, while blue letters are abbreviations of species only occurred in LSW. Black letters indicated abbreviations of co-occurring species in HSW and LSW habitats. NMDS stress = 0.162. Sa, Hs, Rs, As, Kf, Tr, Ha, Ce, Sd, Pe, Hu, Hc, Kc, Sr, Gu, Pa, Hh, Asq, Ns, Av, Sp and Sm stand for *Salsola arbuscula*, *Halocnemum strobilaceum*, *Reaumuria soongorica*, *Alhagi sparsifolia*, *Kalidium foliatum*, *Tamarix ramosissima*, *Haloxylon ammodendron*, *Calligonum ebinuricum*, *Suaeda dendroides*, *Populus euphratica*, *Horaninowia ulicina*, *Halostachys capsica*, *Karelinia capsica*, *Salsola ruthenica*, *Glycyrrhiza uralensis*, *Phragmites australis*, *Halimodendron halodendron*, *Agriophyllum squarrosum*, *Nitraria sibirica*, *Apocynum venetum*, *Suaeda prostrata* and *Suaeda microphylla*, respectively.
(DOCX)

S1 Data (RAR)
