# Supplementary material for: The relative contribution of intraspecific variation and species turnover to the community-level foliar stoichiometric characteristics in different soil moisture and salinity habitats
Source: PLoS One. 2021 Feb 17;16(2):e0246672. doi: 10.1371/journal.pone.0246672 (PMC7888666; doi:10.1371/journal.pone.0246672)
Supplement: S1 Table — (DOCX) [file pone.0246672.s001.docx]

| Habit | Species | Plot | | Average number | |
| --- | --- | --- | --- | --- | --- |
|  |  | HSW | LSW | HSW | LSW |
| Tree | *Tamarix ramosissima* | 2 | 2 | 2 | 4 |
|  | *Populus euphratica* | 10 | 12 | 3 | 2 |
|  | *Haloxylon ammodendron* | 7 | 14 | 2 | 4 |
| Shrub | *Alhagi sparsifolia* | 2 | 9 | 6 | 22 |
|  | *Apocynum venetum* | 6 | 2 | 24 | 8 |
|  | *Halimodendron halodendron* | 2 | 0 | 9 | 0 |
|  | *Nitraria sibirica* | 5 | 2 | 8 | 7 |
|  | *Suaeda dendroides* | 0 | 9 | 0 | 114 |
|  | *Reaumuria soongorica* | 4 | 9 | 3 | 12 |
|  | *Calligonum ebinuricum* | 0 | 3 | 0 | 3 |
|  | *Halocnemum strobilaceum* | 0 | 2 | 0 | 1 |
|  | *Halostachys capsica* | 1 | 1 | 4 | 2 |
|  | *Kalidium foliatum* | 4 | 4 | 2 | 16 |
| Herb | *Suaeda microphylla* | 1 | 0 | 4 | 0 |
|  | *Horaninowia ulicina* | 0 | 8 | 0 | 81 |
|  | *Glycyrrhiza uralensis* | 1 | 0 | 37 | 0 |
|  | *Karelinia capsica* | 3 | 1 | 24 | 36 |
|  | *Salsola collina* | 0 | 11 | 0 | 378 |
|  | *Phragmites australis* | 6 | 2 | 38 | 22 |
|  | *Suaeda prostrata* | 1 | 0 | 13 | 0 |
|  | *Agriophyllum squarrosum* | 0 | 1 | 0 | 8 |
|  | *Salsola ruthenica* | 0 | 2 | 0 | 33 |

**S1 Table.** Plant habit, the number of plots which species appear, the average number of individuals per plot in the two soil moisture and salinity habitats.
