# Supplementary material for: The relative contribution of intraspecific variation and species turnover to the community-level foliar stoichiometric characteristics in different soil moisture and salinity habitats
Source: PLoS One. 2021 Feb 17;16(2):e0246672. doi: 10.1371/journal.pone.0246672 (PMC7888666; doi:10.1371/journal.pone.0246672)
Supplement: S2 Table — *Significant difference in means of P between this study and the others (P<0.001). No significant differences in N were between studies (P>0.05). (DOCX) [file pone.0246672.s002.docx]

| **Data source** | **N(mg/g)** | **P(mg/g)** |
| --- | --- | --- |
| **This study** |  |  |
| Mean | 20.4 | 0.94* |
| SD | 5.8 | 0.43 |
| n | 150 | 150 |
| **Han et al. (2004)** |  |  |
| Mean | 20.2 | 1.46* |
| SD | 8.41 | 0.99 |
| n | 554 | 745 |
| **Reich & Oleksyn (2004)** |  |  |
| Mean | 20.1 | 1.77* |
| SD | 8.71 | 1.12 |
| n | 1251 | 923 |

**S2 Table.** Statistics of leaf nitrogen (N), phosphorus (P) and N:P ratio for all species analysed by this study; Han et al. (2005); and Reich & Oleksyn (2004).

*Significant difference in means of P between this study and the others (*P* < 0.001). No significant differences in N were between studies (*P* > 0.05).
