# Supplementary material for: The relative contribution of intraspecific variation and species turnover to the community-level foliar stoichiometric characteristics in different soil moisture and salinity habitats
Source: PLoS One. 2021 Feb 17;16(2):e0246672. doi: 10.1371/journal.pone.0246672 (PMC7888666; doi:10.1371/journal.pone.0246672)
Supplement: S1 Fig — Red letters are abbreviations for species only occurred in HSW, while blue letters are abbreviations of species only occurred in LSW. Black letters indicated abbreviations of co-occurring species in HSW and LSW habitats. NMDS stress = 0.162. Sa, Hs, Rs, As, Kf, Tr, Ha, Ce, Sd, Pe, Hu, Hc, Kc, Sr, Gu, Pa, Hh, Asq, Ns, Av, Sp and Sm stand for Salsola arbuscula, Halocnemum strobilaceum, Reaumuria soongorica, Alhagi sparsifolia, Kalidium foliatum, Tamarix ramosissima, Haloxylon ammodendron, Calligonum ebinuricum, Suaeda dendroides, Populus euphratica, Horaninowia ulicina, Halostachys capsica, Karelinia capsica, Salsola ruthenica, Glycyrrhiza uralensis, Phragmites australis, Halimodendron halodendron, Agriophyllum squarrosum, Nitraria sibirica, Apocynum venetum, Suaeda prostrata and Suaeda microphylla, respectively. (DOCX) [file pone.0246672.s003.docx]

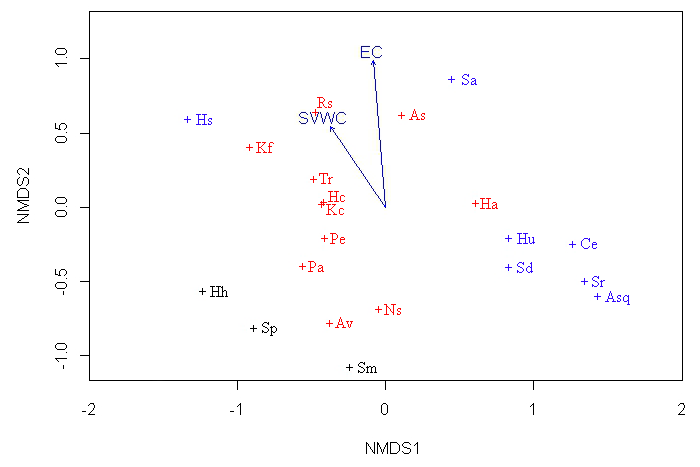


**S1 Fig.** Non-metric multi-dimensional scaling (NMDS) ordination of community composition for plots varying in soil moisture (SVWC) and salinity (EC). Red letters are abbreviations for species only occurred in HSW, while blue letters are abbreviations of species only occurred in LSW. Black letters indicated abbreviations of co-occurring species in HSW and LSW habitats. NMDS stress = 0.162. Sa, Hs, Rs, As, Kf, Tr, Ha, Ce, Sd, Pe, Hu, Hc, Kc, Sr, Gu, Pa, Hh, Asq, Ns, Av, Sp and Sm stand for *Salsola arbuscula, Halocnemum strobilaceum, Reaumuria soongorica, Alhagi sparsifolia, Kalidium foliatum, Tamarix ramosissima, Haloxylon ammodendron, Calligonum ebinuricum, Suaeda dendroides, Populus euphratica, Horaninowia ulicina, Halostachys capsica, Karelinia capsica, Salsola ruthenica, Glycyrrhiza uralensis, Phragmites australis, Halimodendron halodendron, Agriophyllum squarrosum, Nitraria sibirica, Apocynum venetum, Suaeda prostrata* and *Suaeda microphylla*, respectively.
